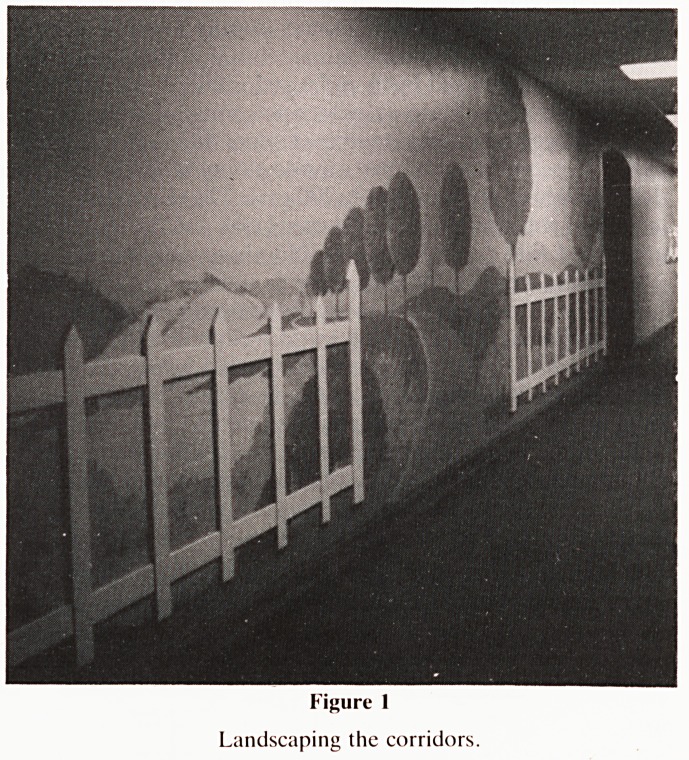# An Arts Co-Ordinator in a Psychiatric Hospital

**Published:** 1990-12

**Authors:** D. S. Allen, S. Hosking

**Affiliations:** Psychiatric Registrar Basingstoke District Hospital, Psychiatric Division, Park Prewett, Basingstoke; Arts Co-ordinator, Basingstoke District Hospital, Psychiatric Division, Park Prewett, Basingstoke


### An Arts Co-ordinator In a Psychiatric Hospital

**D. S. Allen, MB BS MRCGP MRCPsych**
Psychiatric Registrar

**S. Hosking***

Arts Co-ordinator, Basingstoke District Hospital, Psychiatric Division, Park Prewett, Basingstoke.

### Introduction

As patients move out into the community it tends to be forgotten that the old 'asylums' will continue to be the home of many people in the foreseeable future and for many until they die. The environment in which they live is shared, to some extent, by staff and acute patients as well as visitors to the hospital.

Traditionally the decoration of hospitals has proceeded in a rather piecemeal fashion and colour schemes etc. have been chosen by a variety of amateurs (1) and have been dependent on pragmatic factors such as the availability of paint. In addition many of the fine architectural features of the Victorian buildings have been obscured or destroyed for dubious 'practical' reasons.

Although there is some evidence that the bland surroundings can lead to withdrawal and lethargy (2), common sense and humanity dictates that decor should be pleasing to those who have to live with it.

### The post

An arts co-ordinator was appointed to Basingstoke and North Hampshire Health Authority about 3 years ago, initially on a part time and temporary basis. The original brief was to 'commission art for the hospitals' in order to 'brighten things up'. Later advice was sought concerning the colour schemes of several 'routine' painting jobs around the psychiatric hospital, as it became increasingly obvious to management that the use of colour is subject to professioinal expertise (2). The professional background of the appointee is in the creative arts and, for the last fifteen years, in government arts subsidy. The idea, in good British hospital tradition (3), is that money raised from trust funds, charity and private enterprise would be used to finance various projects around the district.

Although a 'District' post, the location of the arts co-ordinator in the psychiatric hospital buildings led to some initial controversy, especially as her brief was seen as somewhat 'frivolous' compared to the other areas perceived to be competing for resources; a common reaction (1). Attitudes changed when the 'self-financing' nature of the job was understood.

### Achievements

Most noticeable to the visitor are the main corridors. Several simple murals have been painted, according to the specification of the designer (Graham Stephenson of Stephenson and Thomas, Winchester), by the hospital painters, who enjoyed the challenge! One includes a picket fence raised from the level of the wall (Fig. 1). Patterns complementing the original architecture have been used and new carpets laid. Plants and pictures, including the beautifully executed original plans of the buildings (with the word 'asylum' covered over to cater for modern day sensibilities!), have sprung up all over. All the money for this has come from patients' trust funds, none from clinical budgets; an important principle (4).

The reception desk has been made more 'user friendly' by removing the glass partition amongst other things and a door has been created into a previously disused courtyard in order to gain access to a newly created 'herb garden'. This is based on a theme of medicinal healing and has been funded by mopney raised by national and local sources. The centrepiece is an amazing reclining seat in multicoloured woods. Last, but not least, quite large areas of grass have been allowed to grow naturally in order to encourage wildlife; an example of an environmentally friendly practice which the N.H.S. has been encouraged to adopt (5).

* Correspondence

**Figure 1**

Landscaping the corridors.

### Conclusions

In our hospital initial comments from patients were positive. Many were appreciative of the fact that something was being done to improve their home/hospital environment. Many staff also, were pleased that the decor had been improved. Although many wards have been 'upgraded' in recent years there is much scope for imaginative improvement. In the current financial climate in the N.H.S. it is unlikely that any but the most basic repairs and redecorations can be carried out using hospital budgets.

The ability of the arts co-ordinator not only to design and implement schemes, but to raise the money to do so as well, has been well proven in Basingstoke and North Hampshire and we would argue that it is a worthwhile contribution to the welfare of patients and staff in our district. Not only do we feel that there is a future for the service locally, but also, that it is something which other districts could well look into.

### REFERENCES

1. DELAMOTHE, T. (1989) Hospital art and its problems. *British Medical Journal,* **298**, 1164–1165.
2. BIRREN, F. (1973) A colorful environment for the mentally disturbed. *Art Psychotherapy.* **1**, 255–259.
3. BARON, J. H. and GREENE, L. (1984) ARt in hospitals. *British Medical Journal,* **289**, 1731–1737.
4. BARON, J. H. (1984) How to beautify your hospital. *British Medical Journal,* **289**, 807–810.
5. GRAY, M. and KEEBLE, B. (1989) Greening the N.H.S. *British Medical Journal,* **299**, 4–5.

## Figures and Tables

**Figure 1 f1:**